# Genomic determination of minimum multi-locus sequence typing schemas to represent the genomic phylogeny of *Mycoplasma hominis*

**DOI:** 10.1186/s12864-016-3284-z

**Published:** 2016-11-23

**Authors:** Aleksey Jironkin, Rebecca J. Brown, Anthony Underwood, Victoria J. Chalker, Owen B. Spiller

**Affiliations:** 1 Colindale, Public Health England, London, UK; 2 School of Medicine, Cardiff University, Cardiff, UK

**Keywords:** Mycoplasma hominis, Typing schema, Genomics, Snp analysis

## Abstract

**Background:**

*Mycoplasma hominis* is an opportunistic human pathogen, associated with clinically diverse disease. Currently, there is no standardised method for typing *M. hominis*, which would aid in understanding pathogen epidemiology and transmission. Due to availability and costs of whole genome sequencing and the challenges in obtaining adequate *M. hominis* DNA, the use of whole genome sequence analysis to provide clinical guidance is unpractical for this bacterial species as well as other fastidious organisms.

**Results:**

This study identified pan-genome set of 700 genes found to be present in four published reference genomes. A subset of 417 genes was identified to be core genome for 18 isolates and 1 reference. Leave-one-out analysis of the core genes highlighted set of 48 genes that are required to recapture the original phylogenetic relationships observed using whole genome SNP analysis. Three 7-locus MLST schemas with high diversity index (97%) and low dN/dS ratios (0.1, 0.13, and 0.11) were derived that could be used to confer good discrimination between strains and could be of practical use in future studies direct on clinical specimens.

**Conclusions:**

The genes proposed in this study could be utilised to design a cost-effective and rapid PCR-based MLST assay that could be applied directly to clinical isolates, without prior isolation. This study includes additional genomic analysis revealing high levels of genetic heterogeneity among this species. This provides a novel and evidence based approach for the development of MLST schema that accurately represent genomic phylogeny for use in epidemiology and transmission studies.

**Electronic supplementary material:**

The online version of this article (doi:10.1186/s12864-016-3284-z) contains supplementary material, which is available to authorized users.

## Background

*Mycoplasma hominis* is an opportunistic human pathogen and resides on the mucosal surfaces of the cervix or vagina in 21 to 53% of sexually mature women; its presence is somewhat lower in the urethra of males. The presence of *M. hominis* is associated with clinically diverse diseases including; urogenital diseases, postpartum fever [28], pneumonia [15], meningitis, post-operative wound infection, post-organ transplant infection [29] and septic arthritis. The capacity of *M. hominis* to cause disease has been proven by induction of preterm labour and development of foetal chronic lung disease following experimental in utero administration of *M. hominis* to pregnant macaque monkeys [19].

Understanding pathogen epidemiology and transmission is important in preventing future infections and comprehending the transmission chains. There is currently no standardised method of molecular typing for *M. hominis* and due to the fastidious growth requirements of Mollicutes, genomic typing is unlikely to be available for routine practice for the foreseeable future. Current discriminatory methods for typing of *M. hominis* to improve understanding of epidemiology of infection and genetic diversity are not in clinical use. Several molecular typing mechanisms have been developed for *M. hominis* including: restriction fragment length polymorphism (RFLP) analysis, amplified length polymorphism (AFLP) [13] and random amplified polymorphic DNA (RAPD) [24]. These techniques have displayed poor reproducibility (RAPD), require specialist equipment, time, and large quantities of biological material to perform the test. Differing typing schemes based on sequence analysis of the *p75*, *p120’* and *vaa* genes do not give concordant results [4, 6, 13, 24]. Multiple locus variable-number tandem-repeat (VNTR) analysis (MLVA) has successfully been used to subtype other *mycoplasma* species. However, the high genetic heterogeneity of *M. hominis* restricted the test’s use to individual studies, and was too discriminatory for large epidemic studies [9].

Multi-locus sequence typing (MLST) analysis of the diversity of housekeeping genes that are considered to be under less selective pressure than other genes have been successfully employed for several bacterial species including Mycoplasmas such as *M. bovis* [26], *M. agalactiae*, *M. hyorhinis* [27] and *M. hyopneumoniae* [16]. Sogaard *et. al* examined six house-keeping gene sequences to investigate evidence of genomic recombination in *M. hominis* and revealed a high degree of variability between these genes [23]. However, the authors did not utilise the data to create a genotyping scheme. The aim of this study was to develop an MLST scheme based on analysis of *M. hominis* genomic sequences to derive the minimum number of genes required to accurately reflect genomic phylogeny.

## Results

### Pan-genome

**Fig. 1:**
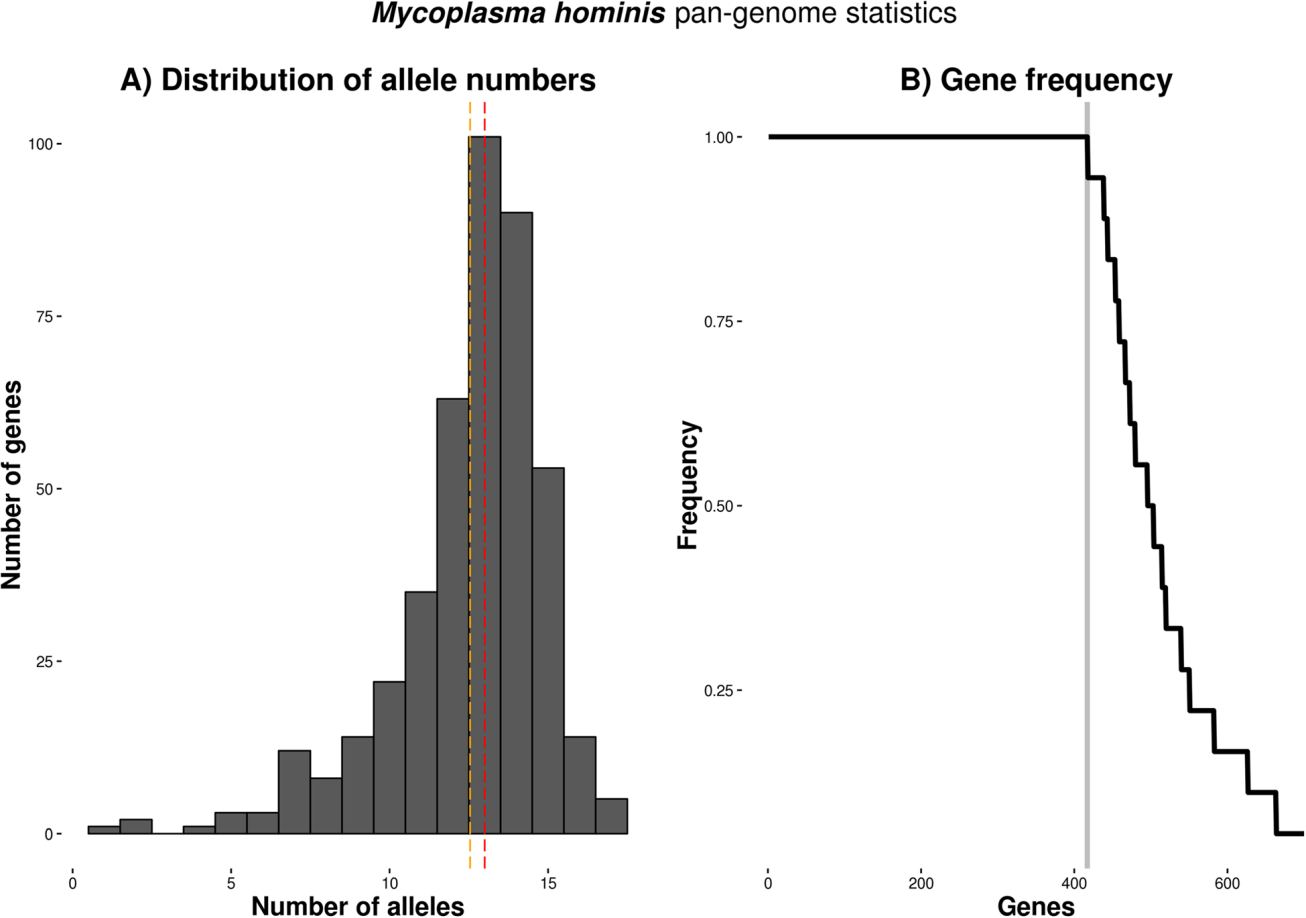
*Mycoplasma hominis* pan-genome statistics: **a**: Allele frequency of the genes in the pan genome is distributed with median 3 alleles (red) and mean 12 alleles (yellow) ; lower peak corresponds to conserved genes (low allele count), higher peak corresponds to genes with many different alleles. **b**: Gene frequency across all samples in the pan-genome. Grey line indicates the core number of genes appearing in all clinical strains and reference strain ATCC 27545 at least once (417)

**Fig. 2:**
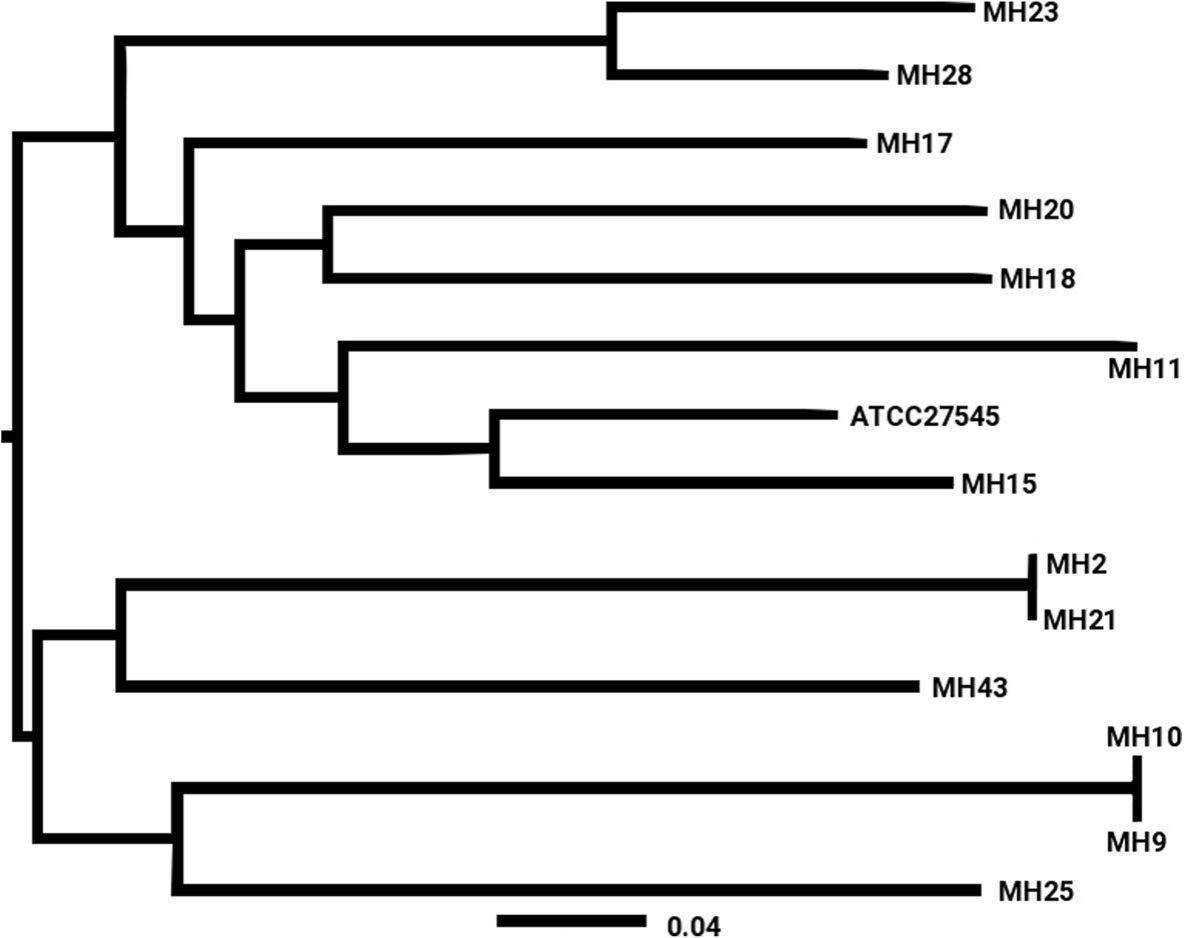
*Mycoplasma hominis* phylogeny: Maximum likelihood phylogenetic tree using only single nucleotide polymorphisms (SNPs) derived using RAxML software shows diversity among the sample set and relatively long branch length

Raw genomic sequence reads from 18 *M. hominis* clinical isolates (Table 1) were assembled and scanned against a database of Hidden Markov Models (HMMs) representing gene coding families constructed using four complete genomes published on NCBI: ATCC 27545 (NZ_CP009652;533 genes), PG21 (NC_013511; 497 genes), Sprott (NZ_CP011538; 524 genes), and AF1 (NZ_CP009677; 531 genes) (see Methods). On average, *M. hominis* pan genome clustering was able to detect 550 (median: 553) genes per sample, which is comparable to the mean number of genes found in the four reference genomes (521). The *M. hominis p*an-genome contained total of 700 genes (Fig. 1) with 417 genes (54.9%; 95% CI: 51.44-58.42) present across all samples and the reference strain ATCC27545 at least once. The shoulders in the pan-genome frequency distribution are likely to correspond to the genes found in the specific phylogenetic clades, Fig. 1(b). The number of alleles per gene family is normally distributed with mean of 15 alleles per gene, Fig. 1(a). This is as expected given the relatively long phylogenetic distance from the reference genome observed in the whole genome SNP tree (Fig. 2). No hyper-variant, large copy number genes, such as transposases and integrases, were detected in the pan-genome data analysis.

**Table 1:** Details of *M. hominis* isolates. Isolates originating from the same patient sample are indicated by boxes

Isolate	Year of Isolation	Isolation site	Further clinical information	Accession Number
MH2	1989	Cerebral spinal fluid	Neonate; haemorrhagic	ERS1204292
MH9	2012	Genital isolate	Isolate from outside C-section wound	ERS1204293
MH10	2012	Genital isolate	Isolate from outside C-section wound	ERS1204294
MH11	2012	Peritoneal fluid	Renal transplant	ERS1204295
MH12	2012	Peritoneal fluid	Renal transplant	ERS1204296
MH15	2004	Blood culture	Post-termination operation	ERS1204297
MH17	2004	Unknown	Unknown	ERS1204298
MH18	1993	Genital	Hepatitis C Virus infection	ERS1204299
MH20	1990	Cervical swab	Unknown	ERS1204300
MH21	1986	Knee aspirate	Unknown	ERS1204301
MH23	2005	Endotracheal secretions	Neonate; 25 weeks gestation	ERS1204302
MH25	2006	Ear swab	Neonate; respiratory distress syndrome; twin	ERS1204303
MH26	2008	Cerebral abscess	Unknown	ERS1204304
MH27	2008	Abdominal pus	Renal transplant	ERS1204305
MH28	2004	Pelvic aspirate	Pelvic haematoma after hysterectomy	ERS1204306
MH29	1989	Pleural fluid	Cardiac failure	ERS1204307
MH43	2013	Spinal tissue	Post-operative; deep tissue infection and superficial irritation; spinal abscess; spinal curdle C5; scoliosis instrumentation	ERS1204308
MH44	2013	Spinal tissue	Post-operative; deep tissue infection and superficial irritation; spinal abscess; spinal curdle C5; scoliosis instrumentation	ERS1204309

### Core-genome MLST

**Fig. 3:**
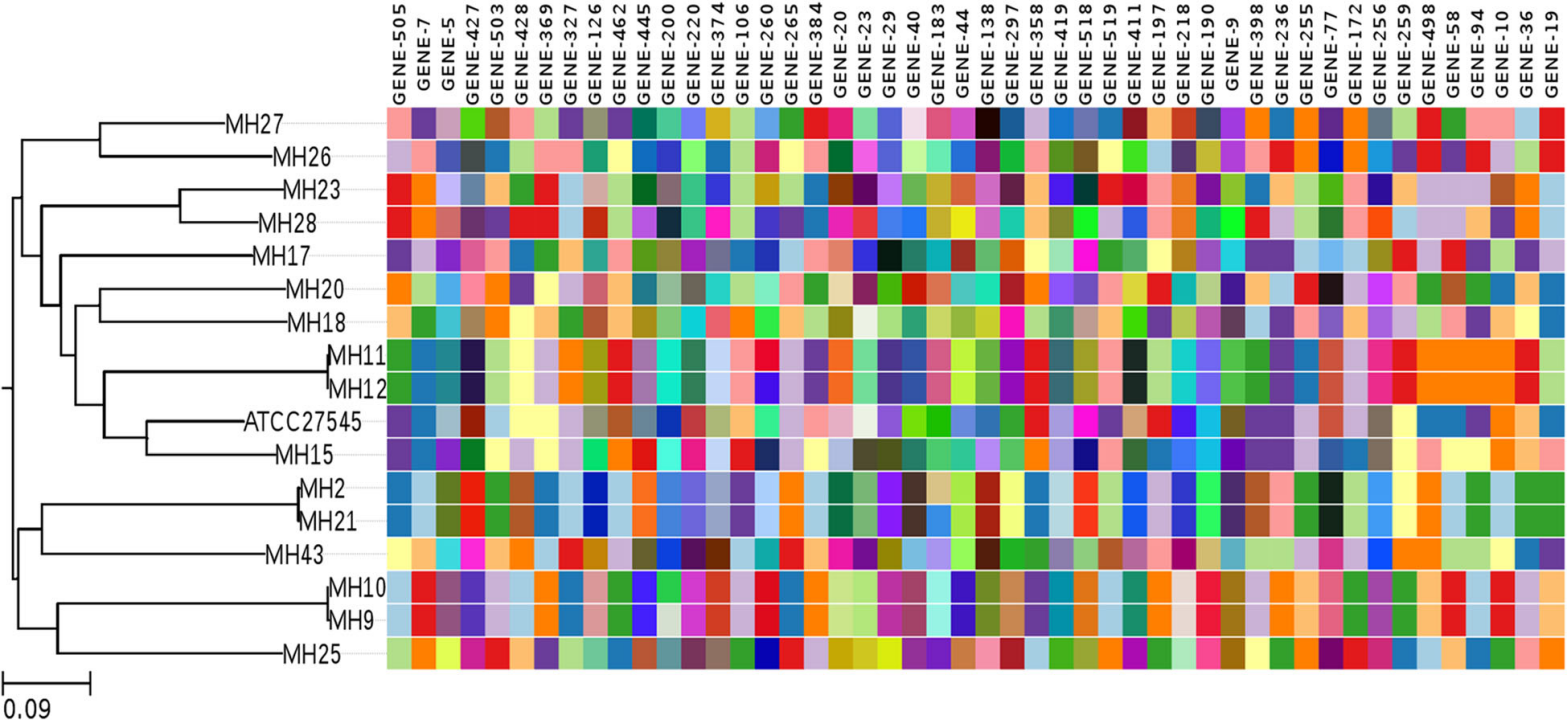
Core gene mosaic for *M. hominis*: Allele mosaic of 48 genes found to be necessary and sufficient for reproducing phylogenetic relationships observed in the maximum likelihood tree constructed with whole genome SNP data. Each colour in a column represent an allele, identical colours in the same column correspond to the same allele. However, colours may be repeated between columns

A subset of genes found across all samples are likely to be highly conserved and carry little phylogenetic signal. Conversely, a small subset of genes could be carrying phylogenetic signal that is similar to the species as a whole, and therefore can be used as a proxy for whole genome evolution. In order to identify genes that carry increased discriminatory potential, leave-one-out analysis was performed. This involved removing one gene at a time from the set of 417 core genes and constructing a phylogenetic tree using the remaining alleles from 416 genes. The resulting phylogenetic tree was compared with the phylogeny derived using whole genome variants (considered the gold standard). Of the 417 genes, 379 genes (88.76%) conferred the same phylogenetic topology as the whole genome tree whilst 48 genes (11.24%) disrupted the phylogenetic relationship of the samples to varying extent. These results suggest that these 48 genes are necessary in reconstructing the correct relationships between the isolates included in this study, Fig. 3(a). To assess the sufficiency of these genes to replicate the topology, a phylogenetic tree was constructed using only these 48 genes (Fig. 3), confirming that these 48 genes were necessary and sufficient for tree reconstruction. These genes are considered the minimum gene set to construct a core-genome MLST scheme for *M. hominis* as they are present in all reference genomes and the 18 sequenced *M. hominis* strains, and are each required to reconstruct the whole genome topology.

### 7-gene MLST schema

Analysis indicated that ^48^C_7_ = 73,629,072 total combinations of allelic profile could be derived from the genomic data of 22 isolates; exhaustive search of a large search space like this is not computationally tractable. Subsets of target genes were selected in order to reduce the size of the search space for further analysis and contained three sets of genes; genes that caused lowest overlap with the whole genome SNP tree in leave-one-out analysis (*n* = 10), manually selected genes for their biological function (*n* = 9), and combination of the above two sets (*n* = 12). Two sets of genomic data from *M. hominis* isolates originating from two single patient samples were included, patient 1: MH9 and MH10, patient 2: MH11 and MH12. These isolate pairs were used to identify which gene sets conferred identical MLST profiles for these lineages. The reduction in the selected target genes resulted in 948 possible combinations of the three gene category subsets. All combinations were analysed for phylogenetic topology closest to that obtained with genomic SNP analysis. Fifteen combinations were identified to produce the highest and identical topological similarity of 0.77 among all tested combinations and three schemas (Table 2) were found to confer the highest similarity to the whole genome SNP tree. All 15 phylogenetic trees classified MH23 and MH28 into a different subtree from the original whole genome analysis. In addition, trees from schemas B and C classified MH17 into a different topological position. Three selected schemas had overall very similar topology to the original whole genome SNP tree representing two general clades and correct bifurcations. Schema A correctly placed MH17 as outer most group to that clade, whereas schemas B and C incorrectly placed the ancestry of MH17. The branch length of the whole genome SNP tree could not be reproduced as shorter sequences with different number of SNPs were used. Overall the three schemas almost completely reproduced the phylogenetic relationship found using whole genome SNP data.

**Table 2:** Three proposed 7-locus MLST schemas for *M. hominis*: The three schemas contain variations of 9 genes that confer phylogenetic relationships similar to those found using whole genome SNPs. Schemas B and C show higher marginal diversity in Hunter-Gaston diversity indicex than schema A. Whereas, schema A shows lowest dN/dS ratio among the three

Gene name	SCHEMA A	SCHEMA B	SCHEMA C	dN/dS (95% CI)	Diversity Index (95% CI)
MHO_4840^*a*^ GENE-20	+	+	+	0.09(0.08-0.1)	0.99(0.97-1.00)
MHO_0720^*a*^ GENE-58	+	+	+	0.27(0.25-0.29)	0.97(0.92-1.00)
*secD* GENE-94	+	+	+	0.07(0.05-0.09)	0.97(0.92-1.00)
*oppA* GENE-138		+	+	0.23(0.21-0.25)	0.96(0.91-1.00)
*argS* GENE-183		+	+	0.07(0.05-0.09)	0.96(0.91-1.00)
*hiss* GENE-236	+	+		0.09(0.06-0.12)	0.96(0.96-1.00)
MHO_1160^*a*^ GENE-265	+		+	0.02(0.01-0.03)	0.97(0.92-1.00)
*tyrS* GENE-428	+		+	0.01(0.01-0.01)	0.97(0.92-1.00)
*dnaG* GENE-519	+	+		0.11(0.09-0.14)	0.99(0.97-1.00)
Mean dN/dS (95% CI)	0.1(0.08-0.11)	0.13(0.11-0.15)	0.11(0.09-0.13)		
Mean Diversity (95% CI)	0.97(0.97-0.98)	0.97(0.96-0.98)	0.97(0.96-0.98)		

^*a*^ gene encoding a hypothetical protein. Gene name relates to annotation in ATCC 23114

### 7-gene *in-silico* PCR

Although full gene schemas can be used for *in-silico* MLST predictions using existing software, e.g. SRST2 [12] it is desirable to have a typing technique based on short (400-600 bp) PCR fragments. A set of primers (Table 3) was designed to minimise polymorphisms in primer binding sites, while maximizing information content required to approximate to whole genome SNP based phylogeny. Tree topologies from sequence products from all schemas were compared to the topologies of trees using full length genes and whole genome SNPs. Schema Ashowed the highest similarity to the topology observed in whole genome SNP tree, similarity score 0.5. Schemas B and C showed lower similarity scores 0.375 and 0.4375 respectively.

**Table 3:** PCR Primers: Proposed PCR primers to be used to amplify the 7 MLST loci

Gene	Forward	Reverse
MHO_4840^*a*^ GENE-20	AATGAACCTATTTATTTTTTGGGTG	AATTTTGAATAAACTGGTATTTCTTTG
MHO_0720^*a*^ GENE-58	CTGCTGCAGCACTTATTGC	GAACGTGATAAAGGAACTACTCA and CGCGATAAAGGAACTACTCA ^*b*^
*secD* GENE-94	GGATGGGATAGTTTTGTGC	ATCTATTTTGATTTGTTGAACTACC
*oppA* GENE-138	GTTACAGTTAAGAGCTTTGATG	ATTGATATAGATCGGTTGGTTC
*argS* GENE-183	CATGGCGGAGATATGATAGA	TTGCATCATTTCCAACTTCTTC
*hiss* GENE-236	CTTTTGAGTCAAGAAATAACTACAT	TTTTTTTCATCTTCATTCAAATAAGCAA
MHO_1160^*a*^ GENE-265	TTTTAGAAGATTTTATTTGCCCACA and TTTAGAAGATTTTATTTGCCCGC ^*b*^	TAAAGTCGCCATTAGCCTG
*tyrS* GENE-428	CTTGCTTCAAGGTTGAGATTTTA	CTTTTGGTTTTATTTTCATTGTGTTG
*dnaG* GENE-519	CTTCCCACTTCATCCTATTTC and ACTTTCCACTTCATCCTATTTCAA ^*b*^	GATTGGCCTGTTTCTTTCATTC

^*a*^ gene encoding a hypothetical protein. Gene name relates to annotation in ATCC 23114

^*b*^ due to SNP in the primer footprint, equal volumes of 2 primers are required to ensure amplification of the target.

### Diversity of MLST genes and determination of synonymous sequence changes

The Hunter-Gaston DI was calculated for each schema and showed that all three schemas have discriminatory power of 0.97 ST per *M. hominis* strain (Table 2). All three MLST schemas are close together in terms of their diversity index indicating that there was no optimal combination of seven genes. Indeed, the schemas only differ by two genes (Table 2). However only scheme A correctly positioned MH17.

Genes under negative selective pressure are more suitable targets for an MLST schema as they are actively conserved in the host, especially where the organism exhibits a high level of genetic diversity. For each of the three proposed MLST schemas the ratio of non-synonymous to synonymous (dN/dS) changes were calculated using the Nei and Gojobori method [18] (Table 2). All genes in all three MLST schemas have a dN/dS ratio of < 1. Schemas A, B and C all contain genes under moderate negative selective pressure: Schema A GENE-58 and GENE-519; Schema B GENE-58, GENE-138 and GENE-519; and Schema C: GENE-58 and GENE-138. The remaining genes have dN/dS ratio below 0.1 indicating strong negative selection. Overall, schema A appears to contain, on average, a more strongly selected gene set (average dN/dS ratio: 0.01), than the other two schemas (average dN/dS ratio: 0.13 and 0.11 for schemas B and C, respectively).

### Seven gene MLST stability

**Fig. 4:**
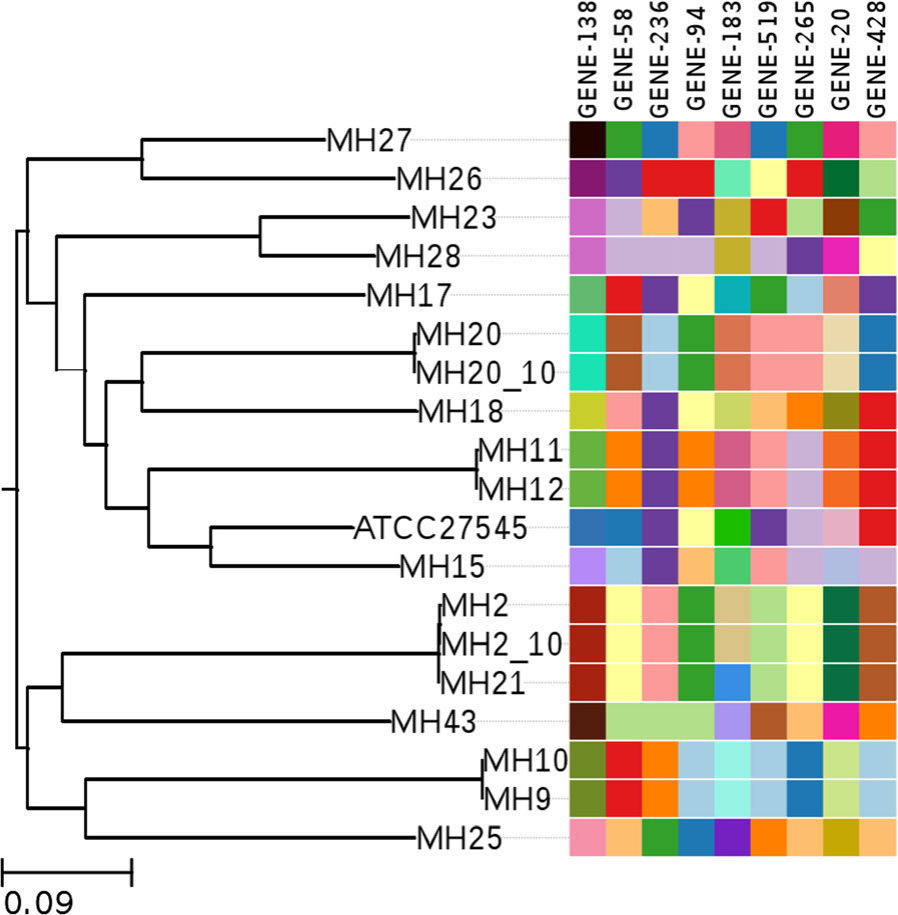
MLST genes stability analysis: Allele mosaic of the 9 genes included from all three schemas shows no variation between alleles for the samples undergone 10 passages (MH2_10 and MH20_10)

Stability of the genes for all three schemas was assessed in two *M. hominis* strains. Whole genome sequencing was performed following short-term passage (10 passages) in liquid culture and compared to the original sequence. None of the genes showed allelic variation in the two strains examined (Fig. 4).

### Recombination analysis

**Fig. 5:**
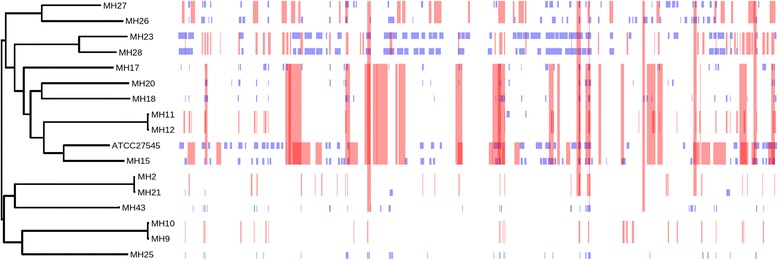
Prediction of recombination in the *M. hominis* isolates: Regions of variation in the genomes of the 18 clinical *M. hominis* isolates and the prototype strain ATCC 27545 which are predicted to have arisen by homologous recombination are shown in the panel on the right. Red blocks indicated recombination predicted to have occurred on internal nodes, blue indicates taxa-specific recombination, isolates are ordered according to the phylogenetic tree displayed on the left

Genomic sequences of the *M. hominis* strains were assessed for predicted regions of variation arising from homologous recombination. Recombination analysis was undertaken using Gubbins. The tree derived using whole genome SNPs and used in leave-one-out analysis was used as a starting tree for Gubbins. Multiple potential areas of recombination were identified across all genomic *M. hominis* data included in the study (Fig. 5). In particular, high levels of recombination were predicted in the phylogenetic clade containing the reference strain ATCC 27454, with multiple recombination events predicted at the same loci.

## Discussion

Assessment of the pan-genome assembled for *M. hominis* revealed 417 core genes. Previous examination of *Mycoplasma* species pan-genome identified a core-genome of only 196 genes (Liu *et al*.); however, this was an inter-species analysis and lower levels of conserved gene than observed in this study is to be expected. Of the entire pan-genome, the core genome represents 55% of all genes. This level of similarity between *M. hominis* strains is in stark contrast to *M. pneumoniae* where over 99% identity has been observed between the genomes [30]. Congruence of the *M. hominis* pan-genome to other *Mycoplasma* species was assessed by comparison to *M. pneumoniae* (strain M129) and *U. parvum* serovar 3 (strain AF222894, ATCC 700970). Pereyre *et al*. have done a similar analysis and presented 247 genes to be orthologous between the three species [20]. No significant hits to any of the genes in the *M. hominis* pan-genome were identified, suggesting that *M. hominis* is a distant relative to these *Mycoplasma* species, sharing little sequence similarity in the coding genes. While Pereyre *et al*. found 247 genes in common; that similarity was based on low percentage identity between the genes. For example, *dnaA* genes from *U. parvum* (UU001) and *M. hominis* (MHO_0040, ATCC 23114) only share 29% identity as reported by BLAST alignment. Whereas, the method used in this study was directed towards high similarity, therefore biased against finding significantly conserved genes. High degree of diversity is concordant with *Mycoplasma* species phylogeny based on 16S rRNA sequences [21]. Kokotovic *et al*. and Busch *et al*. have shown high level of intra-species variability within *M. hominis* by examining clinical isolates using AFLP and RFLP analysis [3, 13].

To determine stability of the proposed genes for the seven gene MLST schemas, sequences were compared before and after short-term passage in two *M.hominis* strains (MH2 and MH20). Genes found to be genetically variable after short-term passage would be unsuitable candidates for an MLST schema. From the set of 48 genes, required to replicate whole genome sequence SNP phylogeny, one gene (GENE-220: *pcrA*) was found to have acquired mutations and was therefore not suitable for use in the MLST schemas. Genomic stability was only assessed in two *M. hominis* strains, it is likely that further instability would be identified upon assessment of a larger number of strains. Sequence heterogeneity has been well documented for *M. hominis* [23] and therefore it is essential that the stability of the genes chosen for the three MLST schemes is further assessed.

Recombination has previously been examined for *M. hominis*; however, only a limited number of genes were examined [23]. Nevertheless, analysis revealed inter- and intra-genic recombination in *M. hominis* and recombination was proposed as a method for the high intra-species variability of *M. hominis*. In addition, examination of *M. pneumoniae, M. genitalium, M. pulmonis* and *Ureaplasma urealyticum* indicated a large number of repeats within the genomes of these organisms suggesting the existence of a large potential for recombination. In concordance with this, a large number of predicted recombination sites were identified in this study. This could be due to the diversity of the isolates from the reference used for mapping. The reference is on average 8500 SNPs from the isolates and each isolate is on average 9100 SNPs from each other isolate, indicating a large degree of diversity. 18 isolates used in this study, although adequate for inferring phylogenetic relationship, do not represent the full temporal and geographic diversity of the *M. hominis* species and future analysis of a larger data set may reveal the true extent of recombination in this species. Currently, with the potential for a large number of recombination events, discriminatory methods that rely on phylogenetic trees have limitations. In this case, the phylogenetic tree is influenced to a greater extent by horizontal transfer of genetic material, rather than vertical inheritance from the parental strains.

The development of MLST schemes has been important both individually and epidemiologically for pathogenic bacteria. At the level of an individual patient, this approach allows discrimination between relapse or persistence and new infection. In this study, *M. hominis* strains originating from the same patient specimen were used to determine gene sets that conferred identical MLST profiles for these strains. Furthermore, identical MLST profiles can be used to identify sexual transmission of *M. hominis* infections or horizontal transmission between mother and baby and may also be used in cases of post-transplant infection. Other methods including MLVA typing of *M. hominis* isolates from two mother-neonate pairs resulted in the identification of identical MLVA types in each case studied, confirming mother-to-child transmission [9]. This study describes MLST schemes with a DI of 0.97 ST per *M. hominis* strain, revealing a genetic heterogeneity among this species.

Genomic sequencing may eventually displace traditional MLST based on multiple gene target PCR and sequencing. However, lack of local/international capability of genomic sequencing technology, specialised culture requirements of mycoplasmas and volume of biomass required to obtain sufficient high quality DNA for analysis is unlikely to lend whole genome sequencing analysis to provide practical clinical guidance for this bacterial species and other fastidious organisms. The major advantage of utilising PCR-based MLST, relative to whole genome sequencing, is the ability to conduct PCR amplification directly on clinical samples. The advent and improvement of metagenomics processes and analysis may eventually supersede this; however, practical use on infections with mycoplasmas and mixed strains is not yet tested. A phylogenetic analysis of a mixed population of *M. hominis* strains would not be possible directly on a clinical sample; however, this may be feasible on infections originating from a clonal *M. hominis* infection

## Conclusions

This study has utilised bioinformatics analysis of *M. hominis* genomic sequence to identify a minimum set of 48 genes required to recapitulate the relationships observed in the whole genome phylogeny of *M. hominis* constructed using SNP data. Following this, three sets of seven genes were identified that could be used to construct an MLST typing schema. Due to availability and costs of whole genome sequencing and the challenges in obtaining adequate *M. hominis* DNA, the use of whole genome sequence analysis to provide clinical guidance is impractical for this bacterial species as well as other fastidious organisms. Furthermore, this study has identified PCR primers and found Schema A to have the highest topological similarity to the phylogenetic tree constructed using whole genome SNPs. The results presented here, provide a novel approach for the development of MLST schemes that accurately represent genomic phylogeny for use in epidemiology and transmission studies.

## Methods

### *M. hominis* strains, Culture and DNA preparation

Eighteen isolates of *M. hominis* from the UK that were submitted to or isolated by Public Health England from 1986 to 2013 from various anatomical locations were included in the study. Isolates were triple cloned on Mycoplasma Agar (Mycoplasma Experience; Surrey, UK) and confirmed as *M. hominis* by amplification and sequencing of *yidC* gene [8]. All isolates were subsequently cultured in Mycoplasma Liquid Medium (MLM; Mycoplasma Experience). All isolates were grown in 100 mL broth culture and the genomic DNA was extracted using the GenElute^TM^ Bacterial Genomic DNA Kit (Sigma; Dorset, UK).

### Next-generation sequencing (NGS)

Genomic sequence data for 18 isolates was obtained using the Illumina Nextera XT sample prep kit (Illumina; Cambridge, UK) and sequenced on an Illumina HiSeq 2500 platform with TruSeq Rapid SBS kits (200 cycles; Illumina) and cBOT for cluster generation (Illumina). Fastq reads were trimmed using trimmomatic 0.32 with the parameters: LEADING: 30; TRAILING: 30; SLIDINGWINDOW: 10:30; MINLEN: 50 [2].

### Genome assembly and pan-genome analysis

Genomic assembly of *M. hominis* was performed using SPAdes v3.6.1 [1] without error correction (−−only-assembler) and 21,33,45,53,65,77,83,93 kmers. For pan genome analysis, four reference sequences (NZ_CP009652.1, NC_013511.1, NZ_CP011538.1 and NZ_CP009677.1) were first clustered together using ggPRO (unpublished). Briefly, coding gene sequences were extracted from the annotated GenBank files for the strains listed above and each gene was checked against the database of hidden Markov models (HMMs) using HMMER v3.2 [11]. A hit was considered significant if the ratio of score over HMM length was greater than or equal to 0.85. Alleles were clustered into the same gene family; new alleles were added to a gene family if they introduced a gap of less than 10% into the gene family alignment. The HMM database was only updated when a new gene or allele was identified. Regions that were not covered by any HMM hits were scanned to check for presence of mycoplasma specific start and stop codons. If they were found, then a putative new gene was assigned in the database. After clustering the four reference sequences, the pan-genome contained 777 genes occurring in at least one reference genome. Each clinical *M. hominis* isolate genome was individually scanned against the pan-genome HMM database using HMMER v3.2 and significant hits (score/hmm length ratio > 0.85) were recorded in the database. FASTA sequence of pan-genome alleles are provided in Additional file 1.

### Phylogenetic analysis

MH44 was excluded from this analysis due to low sequencing yield of the sample. Each remaining sample was mapped against ATCC27545 (NZ_CP009652) using BWA [14] and variants were called using GATK [17] with the following options: −-sample_ploidy 2 --genotype_likelihoods_model BOTH -rf BadCigar -out_mode EMIT_ALL_SITES. The resulting VCF was filtered on minimum depth > 5, AD ratio > 0.9, QUAL score > 40, MQ0 ratio > 0.05 and MQ score > 30. Separate VCFs were combined into a single FastA file and phylogenetic tree with 500 bootstraps was built using RaxML v8.1.17 [25] with GTRGAMMA model, seed 12345 and -f d option.

### MLST gene selection procedure

*M. hominis* genomic sequences were used to develop a minimal MLST scheme. Following pan-genome assembly, alleles of 417 genes were extracted into individual multi FastA files and aligned using Muscle software [7]. Leave-one-out analysis was performed to identify genes required to maintain whole genome sequence SNP phylogeny, whereby one gene was removed at a time and the remaining genes were concatenated into a single multi-fasta file, with one entry per sample. To construct the tree, FastTree [22] with -gtr -gamma -nt parameters was used to reflect similar parameters used for tree construction using RAxML software (see above). The topology of the leave-one-out tree was compared to the whole genome tree using the following formula: *D = S*_*t*_ */S*_*ref*_, where *D* is the similarity score between reference and target trees, *S*_*ref*_ is total number of nodes in a tree, and *S*_*t*_ is the total number nodes such that children of these nodes are the same in reference and target tree. Distance *D,* has strict range (0–1) and represents overall similarity of a target tree to the reference tree, where 0: no similarity and 1: identical trees. This value can be calculated for each internal node for more refined similarity measure. The results were analysed and plotted using custom scripts written for R statistical package. Final MLST allele sequences and sequence types (STs) are found in Additioanl file 2.

### Recombination analysis

Regions of recombination in the whole chromosomes of the *M. hominis* strains were analysed using Genealogies Unbiased by recomBinations In Nucleotide Sequences (GUBBINS) software using default parameters [5].

### Statistics

Diversity of MLST sequence types was assessed using the Hunter-Gaston Diversity Index [10]. A diversity index (DI) of zero indicated no diversity compared to a DI of one indicating complete diversity. The Hunter-Gaston estimate of diversity incorporated a finite sample adjustment. Results included 95% confidence intervals (CI) giving precision to the DI by providing the upper and lower boundaries. Sequences for dN/dS ratios were aligned and translated into protein sequence using NCBI codon (https://www.ncbi.nlm.nih.gov/Taxonomy/Utils/wprintgc.cgi#SG4), and dN/dS ratio was calculated using Biopython. Alleles for GENE-58 contained a number of insertions, which could not be dealt with by Biopython. The insertions were manually deleted from all sequences as they are not accounted by the the Nei and Gojobori model [18].

## Additional files

Additional file 1: FASTA sequence for all pan genomic alleles identified in this study. (ZIP 1488 kb)
Additional file 2: FASTA sequence and sequence type (ST) table for the 7-loci MLST schema. (ZIP 10 kb)

## Abbreviations

AFLP: Amplified length polymorphism
HMM: Hidden markov model
MLST: Multi locus sequence type
PCR: Polymerase chain reaction
SNP: Single nucleotide polymorphism
VNTR: Variable-number tandem-repeat
